# An external sensing system in *Plasmodium falciparum*-infected erythrocytes

**DOI:** 10.1186/s12936-016-1144-6

**Published:** 2016-02-19

**Authors:** Yang Wu, Laura N. Cruz, Tadge Szestak, Gavin Laing, Gemma R. Molyneux, Celia R. S. Garcia, Alister G. Craig

**Affiliations:** Department of Parasitology, Liverpool School of Tropical Medicine, Liverpool, UK; Department of Physiology, Instituto de Biociências, Universidade de São Paulo, São Paulo, São Paulo Brazil

## Abstract

**Background:**

A number of experiments have previously indicated that *Plasmodium falciparum*-infected erythrocytes (pRBC) were able to sense host environment. The basis of this ability to detect external cues is not known but in screening signalling molecules from pRBC using commercial antibodies, a 34 kDa phosphorylated molecule that possesses such ability was identified.

**Methods:**

The pRBC were exposed to different culture conditions and proteins were extracted for 1D or 2D gel electrophoresis followed by Western blot. The localization of 34 kDa protein was examined by biochemical fractionation followed by Western blot. High-resolution mass spectrometric analysis of immune precipitants was used to identify this protein and real-time quantitative reverse transcriptase polymerase chain reaction was used for detecting mRNA expression level.

**Results:**

The 34 kDa protein was called PfAB4 has immediate responses (dephosphorylation and rapid turnover) to host environmental stimuli such as serum depletion, osmolality change and cytokine addition. PfAB4 is expressed constitutively throughout the erythrocytic lifecycle with dominant expression in trophozoites 30 h post-infection. Tumour necrosis factor (TNF) treatment induced a transient detectable dephosphorylation of PfAB4 in the ItG strain (2 min after addition) and the level of expression and phosphorylation returned to normal within 1–2 h. PfAB4 localized dominantly in pRBC cytoplasm, with a transient shift to the nucleus under TNF stimulation as shown by biochemical fractionation. High-resolution mass spectrometric analysis of immune precipitants of AB4 antibodies revealed a 34 kDa PfAB4 component as a mixture of proliferating cellular nuclear antigen-1 (PCNA1) and exported protein-2 (EXP2), along with a small number of other inconsistently identified peptides. Different parasite strains have different PfAB4 expression levels, but no significant association between mRNA and PfAB4 levels was seen, indicating that the differences may be at the post-transcriptional, presumably phosphorylation, level. A triple serine phosphorylated PCNA1 peptide was identified from the PfAB4 high expression strain only, providing further evidence that the identity of PfAB4 is PCNA1 in *P.falciparum*.

**Conclusion:**

A protein element in the human malaria parasite that responds to external cues, including the pro-inflammatory cytokine TNF have been discovered. Treatment results in a transient change in phosphorylation status of the response element, which also migrates from the parasite cytoplasm to the nucleus. The response element has been identified as PfPCNA1. This sensing response could be regulated by a parasite checkpoint system and be analogous to bacterial two-component signal transduction systems.

**Electronic supplementary material:**

The online version of this article (doi:10.1186/s12936-016-1144-6) contains supplementary material, which is available to authorized users.

## Background

Protozoan parasites are capable of withstanding relatively large alterations in their external environments. Signalling is perhaps a primal requirement to respond to stimuli, the signal transduction systems being able to convert an extracellular stimulus into a chemical signal that the cell can sense/perceive and recognize, and can quickly give responses to the changing environment. The malaria parasite has invested in the machinery to transduce signals [1] and a number of experiments, such as those carried out by Cohen et al., have indicated that *Plasmodium falciparum*-infected erythrocytes (pRBC) may be able to sense host environment, where passive transfer of polyclonal sera or purified immune globulin from immune adults into *P. falciparum*-infected individuals resulted in a significant reduction in blood-stage parasitaemia and recovery from clinical symptoms, but also boosted gametocyte production [2]. *Plasmodium falciparum* is considered a micro-aerophilic organism growing in an environment of limited oxygen content 0.5–5.0 %. The malaria parasite is under constant oxidative stress caused by exogenous reactive oxidant species and reactive nitrogen species (RNS) produced by the host immune system and generated by the parasite’s metabolism. Accordingly, parasites have already adapted to cope with the external oxidative stress by adapting to the host environment through superoxide dismutases and thioredoxin-dependent peroxidases, as part of an antioxidant defence system of the pRBC [3]. Additionally, reversible phosphorylation has been shown to play an important role in parasite invasion [4] and intra-erythrocytic growth and development [5], through serine/threonine kinases [6–9].

pRBC are known to be able to activate several signalling pathways in the host: for example, MAPK and PI3 kinases/AKT pathways have been shown to be associated with malaria infection and pathogenesis via cytoadherence [10–12]. It was found that one of NF-kB family members REL-1A (P65), almost completely translocated into the nucleus within 10 min of pRBC-EC interaction, suggesting a direct role for parasite factors in NF-kB activation [13]. Src-family signalling mediated ectophosphorylation of CD36 on endothelium has also been demonstrated, whereby treating HDMEC with a Src-family kinase-selective inhibitor PP1 resulted in a significant reduction of pRBC adhesion in a flow-chamber adhesion assay [14]. However, all of these belong to the host responses to parasite infection, whereas there are only limited numbers of studies about signalling events in parasites themselves, despite the existence of an extensive kinase gene family. For example, signal transduction inside *Plasmodium* has been shown to be a major mechanism to control parasite development [15] with regulation in *P. falciparum* by calcium-dependent protein kinase 7 (PfCDPK7) being reported [16]. PfCDPK1 has been identified as a Ca^2+^-dependent effector that plays a role in microneme secretion during erythrocyte invasion [17]. Mutai and Waitumbi also suggested the existence of *P. falciparum* quorum sensing (the ability to detect conditions of overcrowding), which is more frequently seen in small-molecule signalling pathways in bacteria [18], to keep the parasite population under check [19]. However, the signalling pathways controlling parasite growth and defence have not been well studied. Therefore, research on signalling molecules from the parasite were undertaken to understand their pathways and function in parasite growth, response and defence against the host immune system.

Using a panel of commercial antibodies to several signalling transduction pathways of different species, only one molecule from *P. falciparum* parasites with the appropriate molecular weight was identified. The antibody was against human phospho-I-kappaB-α (IκB), an important component in the NF kappa B (NFκB) pathway of mammals. The NFκB pathway is found in almost all animal cell types, although not in *P. falciparum*, and is involved in cellular responses to stimuli such as cytokines, free radicals, ultraviolet irradiation, oxidized LDL, and bacterial or viral antigens. It mediates stress and innate immunity and plays a key role in regulating the immune response to infection [20, 21].

Activation of NFκB is initiated by the signal-induced degradation of IκBα proteins (a negative feedback regulator). IĸBα functions rapidly and is primarily involved in determining the temporal profiles of NFκB signalling in response to cytokines that serve intercellular communication. Rapid IкBα turnover has been implicated in the high basal NFκB activity in WEHI 231 B immature IgM^+^ B cells [22], primarily via activation of the IκB kinase.

PfAB4 molecule identified in this investigation has several interesting features which could resemble the responses of IĸBα/NFκB in mammalian species. However, there are no NFκB and IκB homologues *per se* in *P. falciparum*, therefore, the identity of the PfAB4 antibody (phosphorylated IκBα antibody)-reacting epitope (called PfAB4 in this study) was one of the first questions. In this paper, the discovery of a sensing system in *P. falciparum* and an investigation into the identity of PfAB4 are reported.

## Methods

### *Plasmodium* culture

*Plasmodium falciparum* isolates used in this study were mainly 3D7 [23], ItG [24] and Dd2 [25], as well as a number of patient isolates PO69, PCM-7, BC12, BC31, and GL-6 recently characterized in our laboratory [26]. Parasites were cultured in vitro in group O^+^ human erythrocytes using previously described conditions [27, 28]. To minimize the effect of antigenic switching in culture, a batch of stabilates was prepared from a post-selection culture and used for no more than three weeks. Mycoplasma contamination of the parasite culture was checked (Universal mycoplasma detecting kit, ATCC, UK). pRBC were regularly synchronized by 5 % sorbitol treatment or Plasmion-gel flotation.

### Sample preparation from infected erythrocytes and immunoblotting

To study the PfAB4 expression profile in the parasites, saponin was added to the parasite culture to a final concentration of 0.05 % and kept on ice for 8 min to lyse the erythrocytes. Following centrifugation at 5000×*g* at 4 °C for 10 min, erythrocyte ghosts were removed and the free parasite pellets were washed twice using RPMI 1640 without serum. The pellet was dissolved in SDS sample buffer (final: 3 % [w/v] SDS, 62 mM Tris–HCl pH 6.8, 15 % [v/v] glycerol) containing 5 % ß-mercaptoethanol), vortexed and concentrated for 5 min at 13,000 RPM to remove any insoluble material, and this was subjected to gel electrophoresis (used as total lysate). This part of pRBC was also further extracted by the fractionation method described by Voss et al. [29] with modifications. Briefly, free parasite pellets were disrupted with ice-cold lysis buffer (20 mM Hepes, pH 7.8, 10 mM KCl, 1 mM EDTA, 1 mM DTT, 1 mM PMSF, 0.65 % Nonidet P-40) and incubated for 5 min on ice. Nuclei were pelleted at 2500×*g* for 5 min, the supernatants were used as parasite cytosol. The nuclear pellet was washed twice in lysis buffer, then re-suspended in 2× pellet volume of nuclear extraction buffer (20 mM Hepes, pH 7.8, 800 mM KCl, 1 mM EDTA, 1 mM EGTA, 1 mM DTT) and put on ice for 30 min with vigorous shaking every 5 min. The extract was cleared by centrifugation at 13,000×*g* for 30 min. The supernatant was used as the parasite nuclear fraction. The insoluble pellet was further washed twice with nuclear extraction buffer, pelleted and used as parasite insoluble fraction. The protein quantity was controlled by using starting material with equal amounts of culture containing the same parasitaemia and haematocrit. Cross-contamination of the nuclear fraction with other fractions was investigated using a number of markers, for example, histone-nuclear localization and localization of other molecules, e.g., HSP90 and PGK should not be in nuclear fraction. All of these fractions were diluted with 2× SDS gel sample buffer. After boiling for 5 min, insoluble material was removed by centrifugation for 5 min at 13,000×*g* and the supernatants were run on Thermo Scientific Precise™ precast polyacrylamide gels for protein electrophoresis using Tris-HEPES running buffer. These fractions were also precipitated by acetone-methanol method [30] and were subjected to two-dimensional (2D) electrophoresis performed as described previously [31]. Briefly, the lysate was solubilized in 2-DE rehydration buffer [8 M urea, 2 M thiourea, 2 % CHAPS, 65 mM dithiothreitol (DTT), and 0.5 % ampholyte pH 4–7 or 3–10]. The sample was vortexed and sonicated on ice ten times for 5 s followed by centrifugation at 15,000×*g* for 10 min. The supernatant was subjected to 2-DE and the isoelectric focusing (IEF) by running on precast Amersham 11 cm pH 3–10 immobiline Drystrip gels using IPG phor IEF Unit (Amersham). The narrow pH precast IPG-strips of pH 3.9–4.9 were from GENETIX. The running programme consists of 10 h for 30 V, 40 min for 200 V, 1 h for 500 V, 4 h for 2000 V and finally 8 h for 8000 V. The voltage was increased gradually until a total of 80,000 vh was reached. The focused strips were equilibrated in 10 ml equilibration solution (50 mM Tris–HCl, pH 6.8, 6 M urea, 30 % glycerol, 2 % SDS) with reducing agent of 1 % DTT for 10 min, and 10 ml equilibration solution with 4.5 % iodoacetamide for another 10 min. The strips were then briefly washed twice with 1× SDS gel running buffer and loaded on 10 or 12.5 % SDS-PAGE gels for second dimension separation. The gels were run at constant current 40 mA in a Laemmli’s buffer system [32] until the dye front reached the bottom of the gel. For Western blot analysis, the gel-separated proteins were transferred electrophoretically to nitrocellulose using glycine-tris-methanol buffer. The nitrocellulose membranes were blocked by 1-hour incubation in 5 % skim milk in TST buffer (0.01 M Tris pH 8.5/0.15 M sodium chloride/0.1 % Tween 20) and washed with TST buffer briefly, then probed with different primary antibodies: anti-phospho-IκB-α (Cell signalling); anti-EXP2 mAb 7.7 (kindly provided by Brendan S Crabb); Anti-HSP90α and HSP90β (Cell signalling); Anti-GAPDH (Enzo); Anti-calcineurin (Abgent); Anti-PGK1/2 (Santa Cruz). All primary antibodies were diluted in Calbiochem Signal Boost immunoreaction enhancer solution 1. Goat anti-rabbit or anti-mouse (as appropriate) IgG (H + L) horseradish peroxidase conjugate (Nordic, 1:2000) was used in enhancer solution 2 to localize antibody-antigen complexes and visualized with Pierce chemiluminescent (ECL2) systems (GE Healthcare).

### RNA extraction and real-time quantitative reverse transcriptase polymerase chain reaction (qRT-PCR)

A TRIzolR Plus RNA Purification Kit (Life Technologies, UK) was used to extract total RNA from the parasite strains using late ring stages at 10 % parasitaemia. RNAs were treated with DNase using a Sigma AMPD1-kit at room temperature for 15 min, then adding stop solution and heating at 70 °C for 10 min. Tetro cDNA synthesis kit (Bioline) was used to generate cDNA according to manufacturer’s instructions with Oligo-(dt)_18_ primer. q-PCR was carried out using Brilliant III Ultra-Fast SYBR Green QPCR Master Mix (Agilent Technologies). Standard curves were generated for each primer set by performing ten-fold serial dilutions of cDNA to produce five different concentrations of starting material. No-RT and DEPC-H_2_O reactions were carried out as negative controls. Details of primers are listed in Table 1. All reactions were performed in triplicate. PCR was carried out in an ABI Prism 7000 (Life Technologies). PCR cycling conditions were 95 °C for 3 min, followed by 40 cycles of 95 °C, 10 s/60 °C, 10 s and a final cycle of 95 °C, 1 min, 55 °C for 30 s, 95 °C for 30 s. Adenylosuccinate lyase (ASL) and seryl tRNA synthetase (STS) were used as internal control genes [33]. Relative quantitation was calculated using the comparative Ct method (ΔΔCt; Applied Biosystems Reference Manual, User Bulletin). Ct values for transcripts of both the reference gene and specific genes were below 30 in all samples. Traditional RT-PCR was also carried out using generated cDNA as template with PF-gene-specific primers and amplification products were ligated directly into the TA cloning vector, pCRI1 (Invitrogen) and sequenced to confirm the products.

**Table 1:** Primer sequences for qRT-PCR analysis

Name	Primer sequences
PCNA1	Forward: 5′ AGTAGGTGATGCTGAAGTAGC 3′
	Reverse: 5′ CCTAAGACAACGACATCTGCT 3′
PCNA2	Forward: 5′ AGGTGTGATAAAAATTGCGTTT 3′
	Reverse: 5′ TCGTCAGCACTTGATTGTTC 3′
EXP2	Forward: 5′ AGATGGTCACGTATGTGGTG 3′
	Reverse: 5′TGGTTTGGATTCTACGGCAT 3′
HSP86family	Forward: 5′ ACCAAGAAGAGGAGAAGTTGG 3′
	Reverse: 5′ GCTCTTTGTTCTTCTGCACT 3′
ANP32	Forward: 5′ ACAGACCTTAGAAAATATCCCATCA 3′
	Reverse: 5′ TGGTTTCCACCTATTTCAAGGG 3′

### Immunoprecipitation (IP) and co-IP using AB4 antibody with Dynabeads^®^

This is a powerful technique to specifically pull down molecules reacting directly with antibodies (IP) and to indirectly capture proteins that are bound to a specific target protein (co-IP). 3D7-infected and uninfected erythrocytes (used as control) were disrupted by saponin lysis at 4 °C and washed with RPMI medium without serum. The parasite pellet was then disrupted with IP lysis buffer (50 mM Tris–HCl pH 7.4/150 mM NaCl/0.5 % NP-40/1.5 mM MgCl_2_) by vortexing and centrifuged at 13,000×*g* for 20 min at 4 °C. Supernatants were cleaned using pre-immune rabbit sera to remove any non-specific reaction and then incubated with primary antibody against PfAB4 diluted 1:200 for overnight under gentle rotation (15 rpm) at 4 °C. Dynabeads^®^ protein A or G (Invitrogen) washed three times in cell lysis NP-40 buffer were added into the lysate-antibody mixture and incubated at room temperature for 2 h with gentle rotation. Beads containing antibody-antigen complexes were washed three times in NP-40 buffer using magnetic isolation and reacted proteins were eluted from beads by adding 2D gel-electrophoresis buffer, vortexed, centrifuged and subjected to 2D gel electrophoresis.

### Protein in-gel digestion, phosphopeptide enrichment, nanoflow LC/MS/MS analysis and database searching

Spot picking of interesting proteins in 2D gels was guided by immunoblot images on duplicate gels. Immunoprecipitated samples were stained with Coomassie blue and the most abundant spots were picked. In-gel digestion was performed as described below: the excised protein spot was put into an Eppendorf Ultra-Pure 1.5 ml centrifuge tube. The band was then cut into 1-cubic mm cubes and rinsed twice in 200 µl MilliQ water for 15 min. The gel slices were dehydrated by the addition of 100 µl of 50 % (v/v) acetonitrile/water and incubated at room temperature for 10 min. One-hundred µl of ammonium bicarbonate (50 mM) was then added to each sample and incubated again at room temperature for 10 min. These last two steps were repeated. After removal of the ammonium bicarbonate, 10 µl of sequence grade trypsin (Promega Southampton, UK) (10 µg ml^−1^ in 50 mM ammonium bicarbonate) was then added to the gel fragments and incubated at 37 °C for 15 h (overnight), after which the supernatant was removed and kept. Twenty µl of 70 % acetonitrile (v/v in water) was added to the gel and incubated for 10 min at room temperature. The supernatant was then removed and pooled with the previous supernatant. The combined supernatant was dried in a speed-vac, re-suspended in 12 µl of 0.1 % formic acid.

The trypsin-digested peptide sample or phosphopeptides, enriched by Magnetic Titanium Dioxide Phosphopeptide Enrichment kit (Pierce) according to manufacturer’s instructions, was centrifuged at 13,000×*g* for 15 min to remove any insoluble material. The supernatant was placed in a new tube and diluted using 0.1 % (v/v) formic acid according to loading requirements. For LC-MSMS analysis: peptides were initially separated by reverse-phase chromatography using a DIONEX UltiMate™ 3000LC chromatography system. For MS proteomics, 10 µl of peptides were injected onto a C18 reverse-phase column [2 µm particle size (100), 75 µm diameter × 150 mm long] at nanoflow rate (0.3 µl min^−1^) and separated over linear chromatographic gradients. The gradients employed for chromatographic separation were composed of buffer A (2.5 % acetonitrile: 0.1 % formic acid) and buffer B (90 % acetonitrile: 0.1 % formic acid). For in-gel proteolysis we employed a 60-min linear chromatographic gradient. Following chromatographic separation, MS analysis was performed on an LTQ Orbitrap Velos mass spectrometer using Xcalibur (version 2.1) software (Thermo Scientific, UK). Ions were scanned between 350–2000 *m/z* in positive polarity mode. The ion-trap operated with CID MS/MS (with wide band activation) on the 20 most intense ions. Dynamic exclusion was enabled to avoid repeatedly selecting intense ions for fragmentation and this was set at 500 with an exclusion duration of 20.0 s. Charge states of 1 were rejected. The minimum MS signal threshold was set at 500 counts and the MS/MS default charge state was 2 with a 1.2 *m/z* isolation width, normalized CID at 35 V and an activation time of 10 min. The resulting MS/MS spectra were submitted to Proteome Discoverer (Thermo Scientific, UK) version 1.2. Searching was against NCBI *P. falciparum* and human databases separately and was performed using fixed carbamidomethyl and variable phosphorylation modifications. Peptide tolerance was set at 0.5 Da, MS/MS tolerance was set at 0.1 Da. Phosphorylated protein identities were considered significant if the protein score was over the 95 % confidence limit and at least one phosphorylated site was unambiguously identified when a phosphorylated residue existed (matched) y- or b-ions in the peak lists of the fragment ions [providing evidence of observed neutral loss of H_3_PO_4_ from the precursor or identified intact phosphorylated residues of serine (pS), threonine (pT) and tyrosine (pY)]. If the protein score reached a significant level but the ion score of phosphorylated peptide was under the 95 % confidence limit, these were referred to as potential-phosphorylated proteins.

## Results

Using ten commercial antibodies to five different signalling transduction pathways, only one positive molecule from *P. falciparum* parasites was identified with an anti-mammalian phospho-IĸBα antibody, with a molecular weight (34 kDa) (Additional file 1). This molecule was named PfAB4 and further characterization of PfAB4 in several *P. falciparum* laboratory parasite lines was undertaken.

**Fig. 1:**
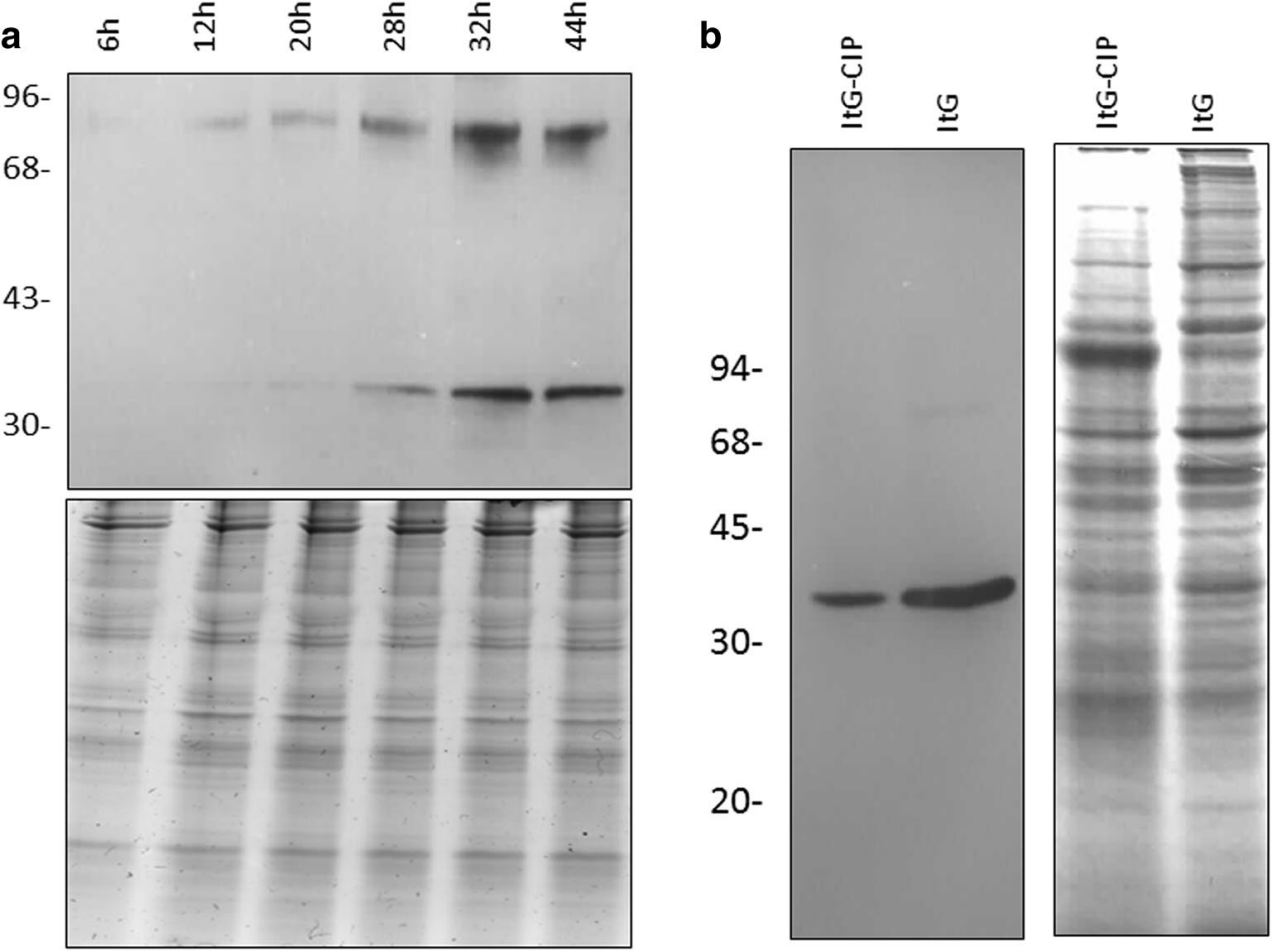
Synchronized stage specific ItG parasite lysates at 14, 24, 36 and 46 h after parasite invasion were analysed by Western blot using AB4 antibodies. PfAB4 expressed throughout parasite lifecycle with expression peak between 28 and 32 h. A 90 kDa protein also reacted with AB4 antibodies. The* lower part* is the Coomassie blue stained gel image used as loading control (**a**). *Panel* **b** shows parasite strains with CIP in vitro treatment, which attenuated the signal indicating the phosphorylation state of PfAB4 proteins

According to PlasmoDB, there is no IκB and other components of the NFκB pathway in *Plasmodium* parasites. By immune blot using this antibody, we found that PfAB4 is a 34 kDa protein expressed at the asexual stage of *P. falciparum*, the expression peak starts at 30 h after invasion and lasts to late schizont stages (up to 44 h); at the early ring stage the expression level was low (Fig. 1a). Calf intestinal alkaline phosphatase (CIP) treatment of pRBC lysates from several parasite strains decreased their expression of the PfAB4 epitope, indicating that PfAB4 is a phosphorylated protein (Fig. 1b). There is also a phosphorylated 90 kDa protein reacting with AB4 antibodies but lacking stimulation responses, therefore work in this paper mainly focused on the 34 kDa PfAB4 protein.

**Fig. 2:**
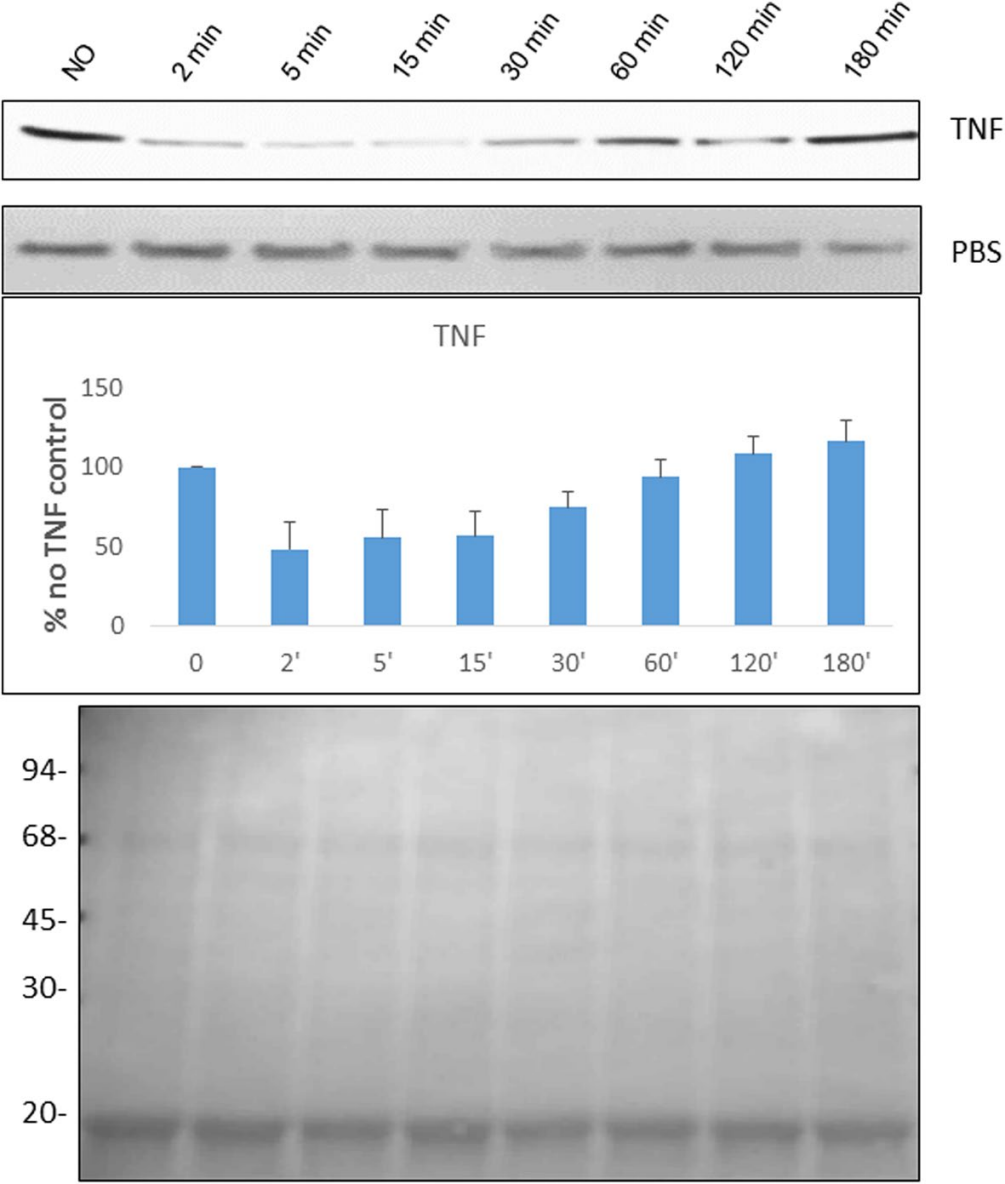
ItG parasites at mid-trophozoite stage were exposed to TNF (1 ng ml^−1^) treatment for 2, 5, 15, 30, 60, 120 and 180 min. PBS mock treatment was performed as a control. The expression of PfAB4 from total cell lysates was determined by Western blot using AB4 antibodies (*upper panel*). The *middle panel* shows the quantification of PfAB4 level from three independent experiments by scanning autoradiographs. The *lower panel* shows a Coomassie blue stained image as a loading control

The second feature is the inducible nature of PfAB4 epitope. This was seen as a rapid turnover and early (immediate) response to several stimuli such as osmolality change, serum depletion/addition and cytokine stimulation. This was a reversible process which is recovered within several hours. For example, TNF induced a loss of the PfAB4 signal, presumably due to dephosphorylation/degradation, in several parasite strains (Fig. 2). This phenomenon was detectable after 2 min of treatment with TNF, while at 60-min incubation the level of this epitope was almost back to the original (0 min) signal. The response to osmolality is shown in Additional file 2.

**Fig. 3:**
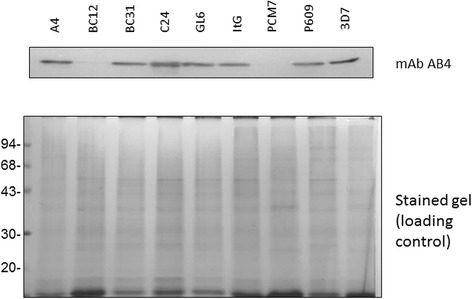
Different parasite strains have different expression levels of PfAB4. The total protein lysates were extracted from ten well-synchronized parasite strains and analysed for PfAB4 expression level. Dd2, BC12 and PCM7 were repeatedly identified as* low* expression strains. The* lower panel* shows the Coomasie blue stained image used as a loading control

The third feature is that different parasite strains have different expression levels of the PfAB4 epitope, with the levels ranging from high expression to almost absent expression (Fig. 3). To confirm that this profile is not caused by the variation in stage-specific expression, the expression levels of PfAB4 in both high expression and low expression parasite strains were measured at time points throughout intra-erythrocyte development cycle (IDC). The strains of Dd2, BC12 and PCM7 showed consistently low expression at all stages, while ItG, 3D7 and A4 demonstrated high PfAB4 expression during the IDC. Biochemical fractionation showed that the PfAB4 protein in low expression parasite strains, such as Dd2, was only detected in the parasite cytosol and no changes were seen during IDC development (Additional file 3).

**Fig. 4:**
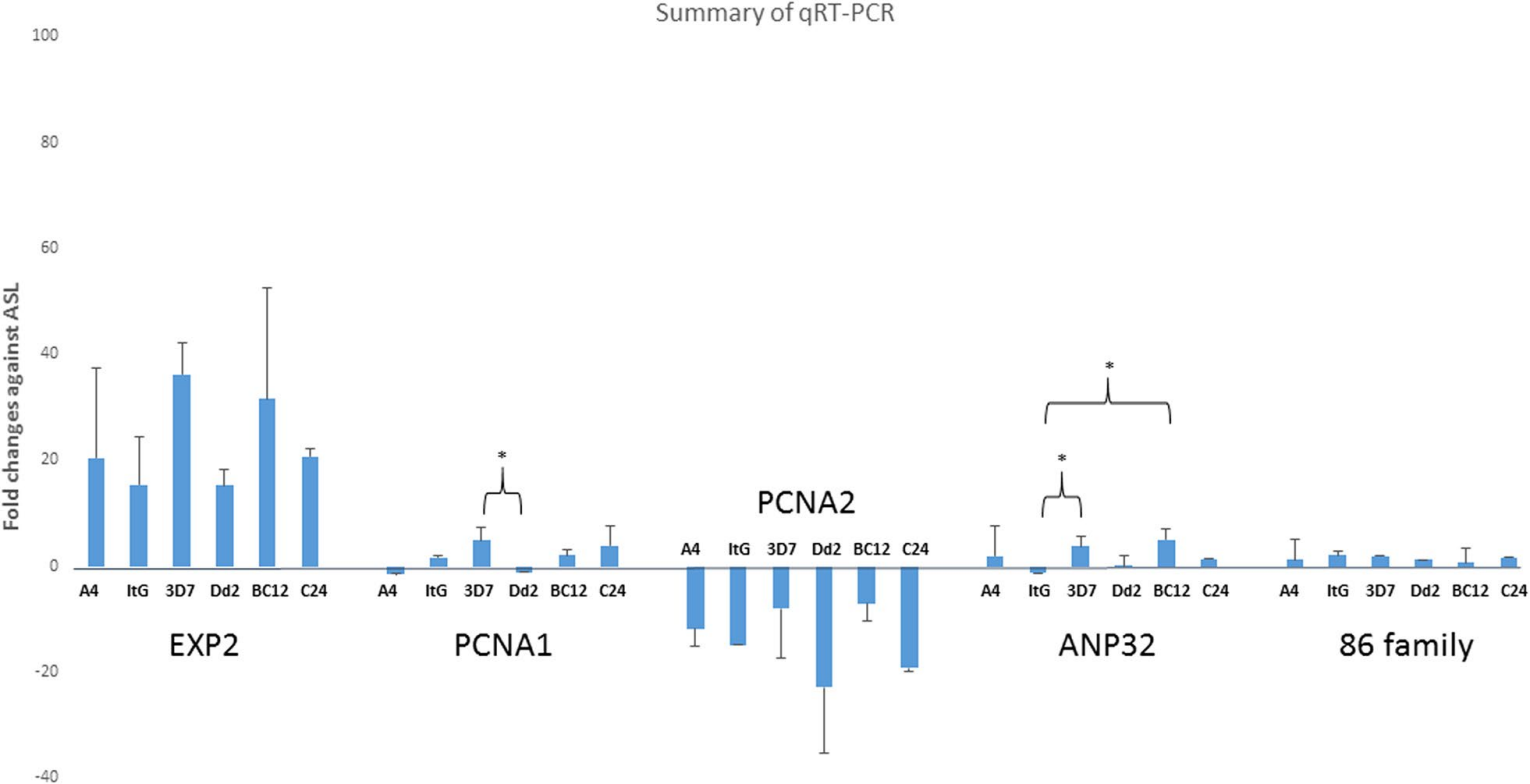
RT-qPCR analysis of selected parasite strains for transcript levels of five candidate genes. Three independent qPCR runs were analysed using the 2^−ΔΔCT^ method. Data are presented as fold changes against an internal reference gene ASL. The results of Student’s *t* tests are indicated as *P < 0.05; *t* tests compared the same gene among different strains

To understand if this difference was at the transcriptional level conventional RT-PCR was carried out for the strains with known PfAB4 expression level, but the results were inconsistent, therefore, qRT-PCR was carried out to measure the transcript abundance of the candidate genes (based on preliminary MS data) from different parasite strains with known PfAB4 epitope levels. Three independent RT-qPCR experiments were carried out for six parasite strains and five target genes, all results were normalized using internal control and represented as fold change. The most abundant transcript was EXP2, and the least abundant was PCNA2. All strains showed variable patterns of transcription of the genes being tested, but these were not co-incident with known protein levels for PCNA1 and EXP2 (Fig. 4). PCNA1 and EXP2 from both Dd2 and ItG strains (low and high PfAB4 expressers, respectively) have been cloned and there were no sequence differences in *pcna1* between the two strains, although there was a deletion mutation difference in *exp2* between Dd2 and ItG strains.

**Fig. 5:**
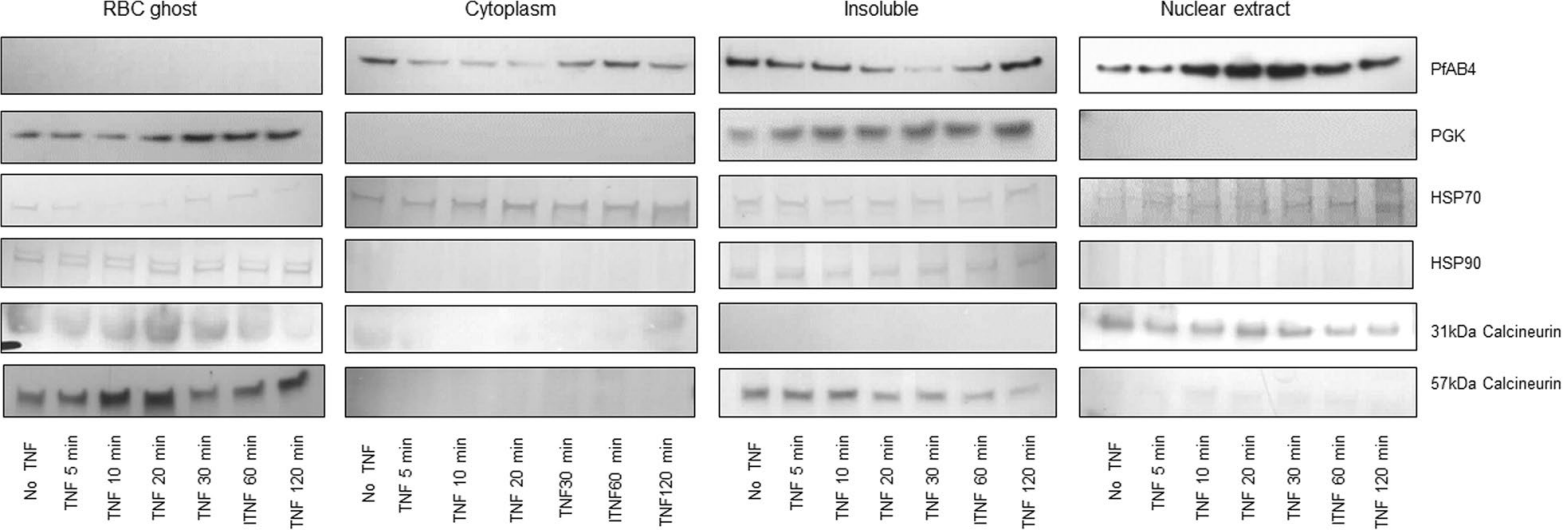
ItG pRBC were treated with TNF (1 ng ml^−1^), at the given a time points, followed by cell fractionation using combined methods of saponin lysis and detergent extraction. PfAB4 and other candidates’ expression and localization were revealed by Western blot. PfAB4 is localized in parasite cytoplasm, nuclear and insoluble fractions. In response to TNF treatment there was a transient shift from cytosol and insoluble compartments to the nucleus within 20–30 min. PGK and calcinurin also showed responsiveness to TNF treatment

To view better the expression profiles of PfAB4 under TNF stimulation in terms of localization, a combined method of saponin lysis and detergent nuclear extraction was used to separate ItG proteins into pRBC ghost, parasite cytosol, nuclear, and insoluble fractions. These four fractions were then subjected to either 1D SDS-PAGE or 2D gel electrophoresis followed by immune-blot using the AB4 antibody. As indicated by fractionation, 1D SDS-PAGE and immuno-blotting (Fig. 5), PfAB4 is localized both in parasite cytoplasm and nucleus. Interestingly, this epitope was able to shift between the cell compartments under TNF treatment such that for 10–30 min it migrated from cytosol and insoluble fractions to the nucleus. At 60 min it was seen to accumulate back in the cytosol and insoluble fractions. No PfAB4 was detected in the pRBC ghost fraction.

**Fig. 6:**
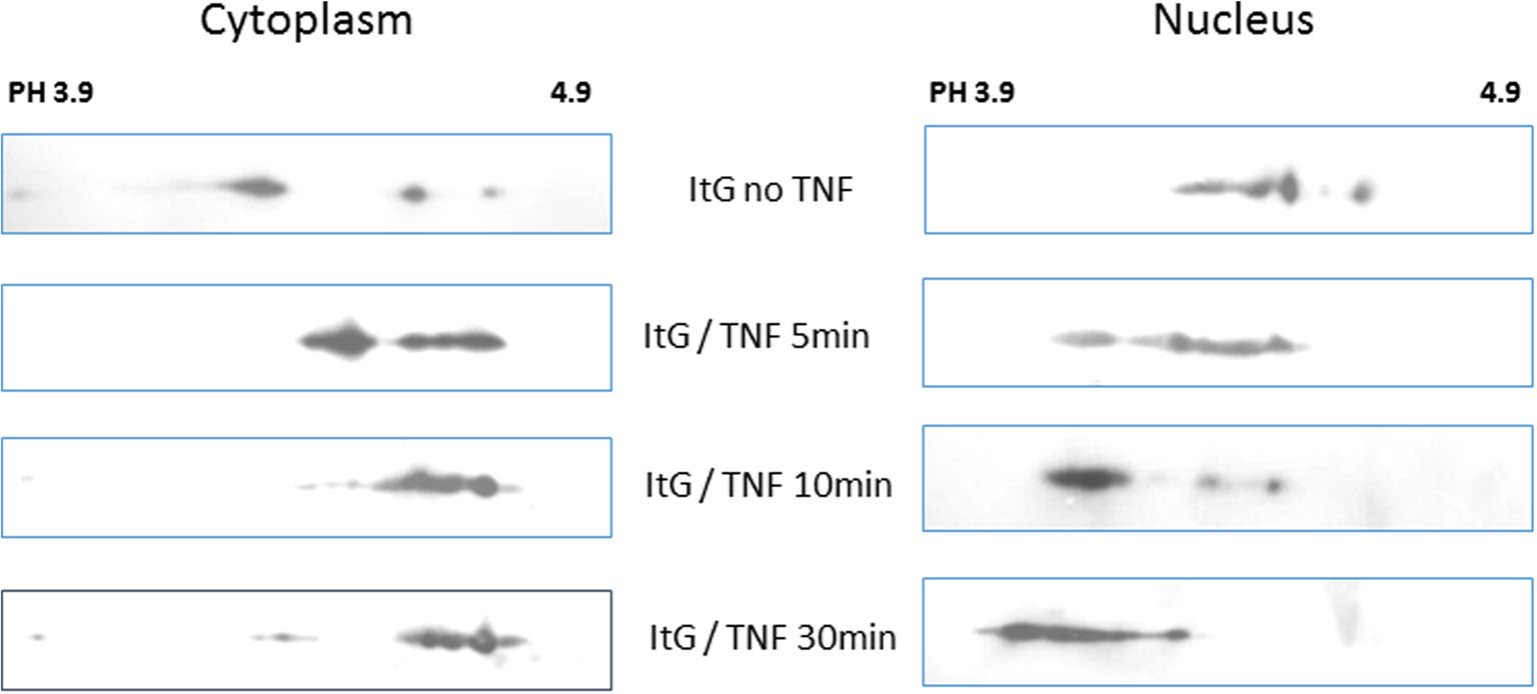
Proteins from cytoplasmic and nuclear fractions of ItG pRBC before or after TNF (1 ng ml^−1^) treatment at the four time points were subjected to 2D gel electrophoresis using narrow pH (3.9–4.9) immobiline Drystrips. PfAB4 expression was revealed by Western blot, showing different patterns of migration in the nucleus and cytoplasm over time

In response to TNF stimulation, PfAB4 molecules not only shifted from one compartment to another, but they also changed their isoelectric point (PI). To further analyse these changes, 2D gel strips with a narrow pH range of 3.9–4.9 were used to separate PfAB4 isoforms at several time points after TNF treatment. Shown in Western blots in Fig. 6, PfAB4 isoform shifting in the nucleus was from pH 4.9–4.5, moving from basic to acidic direction, while in the cytosol, the PI of PfAB4 isoform showed minor shifts within the range pH 4.5–4.9, from acidic to basic direction (Fig. 6).

**Fig. 7:**
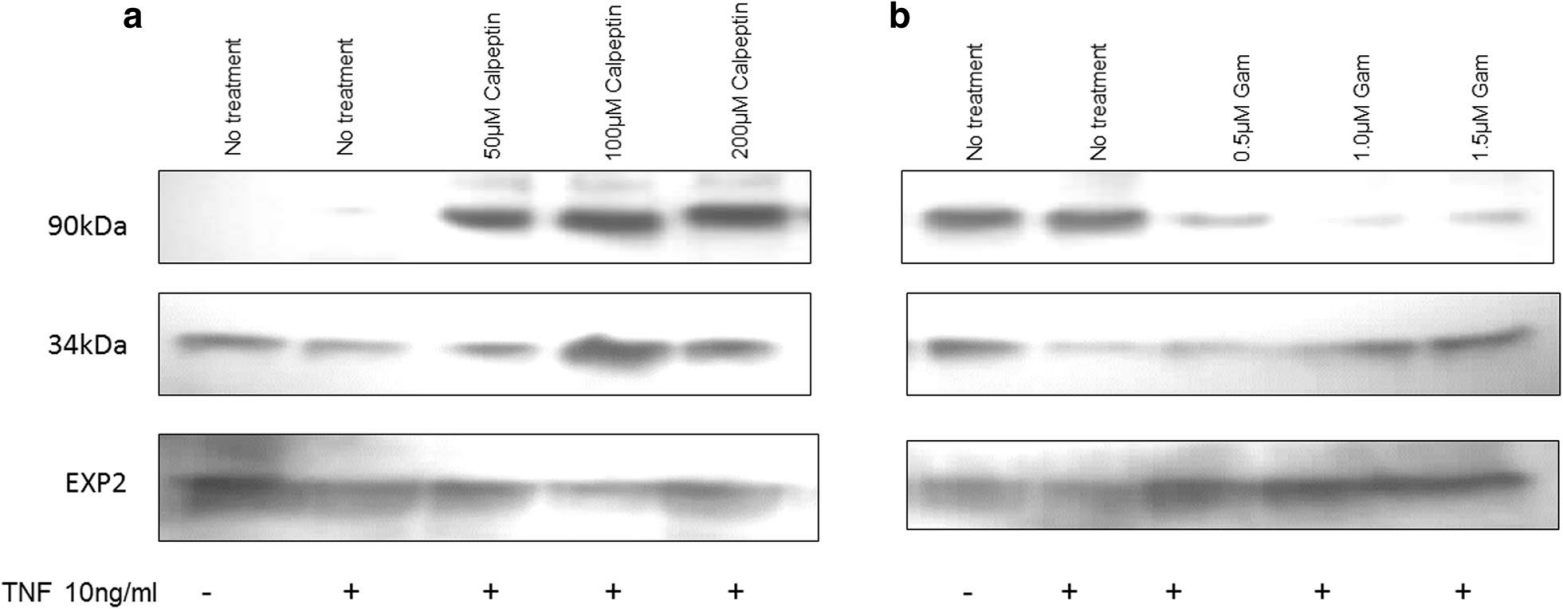
Treatment with different inhibitors, Calpeptin and Gambogoc Acid. **a** ItG early trophozoite stage pRBC were treated with/without calpeptin, a calpain inhibitor for 1 h before TNF stimulation, and the expression of PfAB4 and a 90 kDa protein reacting with AB4 antibodies measured. The 100 µM calpeptin treatment caused a block of PfAB4 degradation. **b** ItG early trophozoite stage pRBC were treated with/without Gambogic acid (Gam), a HSP90 inhibitor overnight before TNF stimulation. The expression of PfAB4 and a 90 kDa protein reacting with AB4 antibodies was measured. Gam 0.5, 1.0 and 1.5 µM inhibited the 90 kDa protein expression and reduced the effect on PfAB4 caused by TNF

One-hundred and 200 µM calpeptin (a calpain inhibitor) treatment 1 h before TNF activation caused a block in the degradation of PfAB4 after stimulation with TNF (Fig. 7), suggesting that changes in expression of PfAB4 might be the result of a ubiquitin-independent pathway and subsequent degradation by a proteasome complex, as previously suggested in other systems by inhibition experiments with peptide aldehyde inhibitors of the proteasome, such as calpain inhibitor I [34, 35]. Gambogic acid is an important anti-cancer drug candidate, a natural product inhibitor of HSP90, which inhibits heat shock protein 90 and down-regulates TNF/NFκB. It has been shown that Gambogic acid physically binds to HSP90, inhibiting its ATPase activity and leads to degradation of HSP90 client proteins (i.e., Akt, IKK) in HeLa cells [36]. In this study, overnight pre-treatment with Gambogic acid (0.5, 1.0 and 1.5 µM) not only inhibited the expression of 90 kDa AB4-associated protein, but also abrogated the effects of TNF on 34 kDa PfAB4 expression (Fig. 7) suggesting that AB4 antibody recognized 90 kDa protein could be a member of HSP90 family and that 34 kDa PfAB4 may be associated with HSP90.

There were a number of proteins with sizes around 34 kDa as seen by staining and immunoblots with different antibodies, and intensive methods have been used to biochemically separate PfAB4 protein using, for example, different gel concentrations, buffer systems and gel types. The best and the most reproducible technique was with 2D electrophoresis separating immune precipitants from IP/Co-IP by AB4 antibodies. This allowed the specific separation of the target proteins and their associates. The 34 kDa reaction spot contains in very close proximity or overlapping, a mixture of PCNA1 and EXP2 along with a small number of other inconsistently identified peptides. A 90 kDa protein was also immuno-precipitated/co-immunoprecipitated with IDs of HSP86 and HSP70 (Table 2).

**Table 2:** Mass spectrometry identifications of AB4 immno-precipitated material

Accession No.	Description	MW	PI	Score
The low molecular weight 34kDa PF-proteins from IP with AB4 antibody				
gi3021540	Exported protein 2	32.9	4.87	455
gi124513948	Proliferating cell nuclear antigen 1	30.9	4.78	167
gi160537	Surface protein	27.6	5.12	148
gi111034851	L-lactate dehydrogenase	34.0	7.55	143
gi296005038	14-3-3 protein	30.2	4.92	140
gi124512500	DNA/RNA-binding protein α	27.2	10.58	96
gi124512686	Proteasome beta-subunit	30.9	6.44	80
gi124806227	Proliferating cell nuclear antigen 2	30.1	4.68	46
The high molecular weight 90kDa PF-proteins from IP with AB4 antibody				
gi124506906	Heat shock protein 70	72.3	5.31	339.91
gi124506155	Myo-inositol 1-phosphate synthase, putative	69.1	7.4	119.2
gi124511730	Heat shock protein 86	86.1	5.01	109.85
gi124512406	Heat shock 70 kDa protein homologue	71.5	6.09	62.36
gi124513604	Kelch protein, putative	83.6	5.67	164.86
gi124512982	Vacuolar ATP synthase, catalytic subunit a	68.6	4.98	117.28
gi258597535	Chaperonin, cpn60	81.4	5.11	42.84
gi124802912	Erythrocyte membrane protein, putative	80.2	4.98	113.77

**Fig. 8:**
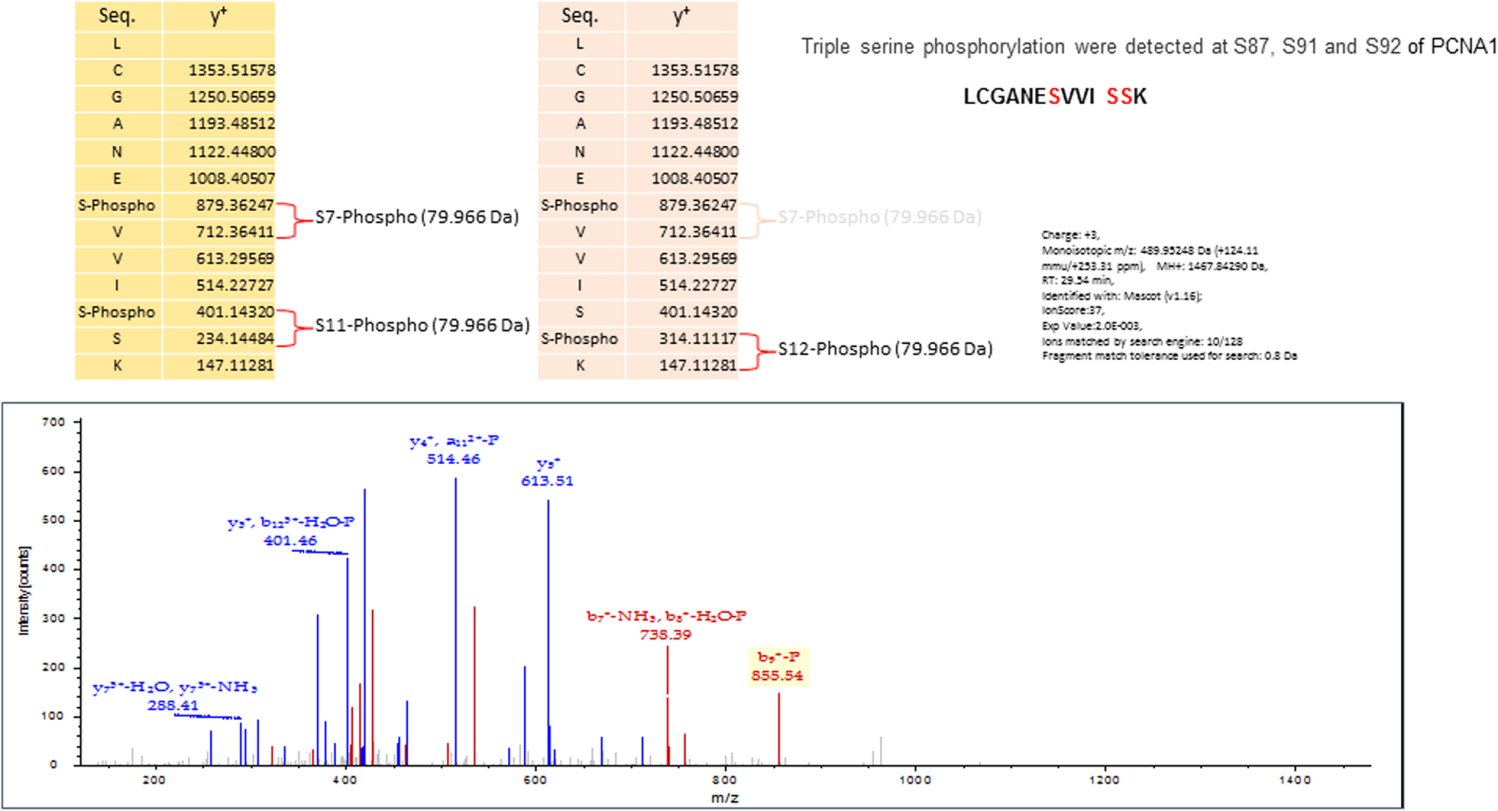
The 32–35 kDa region (covering the PfAB4 proteins) of ItG pRBC from 1D SDS-PAGE were excised and in-gel digested with trypsin. The phosphopeptides were enriched using Pierce Magnetic Titanium Dioxide kit and subjected to Oribitrap mass spectroscopy. This enabled the identification of triple phosphorylation sites in the PfAB4 protein at serine 87, serine 91 and serine 92, which is the PfPCNA1 peptide: LCGANEsVVIssK

To confirm the phosphorylation states of the target protein and to identify the phosphorylated peptides in target proteins, a Titanium Dioxide Phosphopeptide Enrichment kit (Pierce) was used to pull down the phosphorylated peptides after 1D SDS-PAGE separation and trypsin in-gel digestion, from both low and high PfAB4 expression parasite strains, Dd2 and ItG. The resulting trypsinized peptides were subsequently subjected to Orbitrap MS analysis, with the results shown in Table 3. The phosphorylated peptides of PCNA1 were only found in ItG and not Dd2, and there were no EXP2 peptides. Therefore the *Plasmodium* protein recognized by antibody to human phospho-IκB-α antibody (AB4) is likely to be PfPCNA1. These experiments enabled us to identify triple phosphorylation sites in the PfAB4 epitope at serine 87, serine 91 and serine 92 in PCNA1 (Fig. 8).

**Table 3:** Mass spectrometry identifications from phospho-peptide enrichment

	Dd2 before enrichment	Dd2 after enrichment	ItG before enrichment	ItG after enrichment
34 kDa/no treatment	Heat shock 70 kDa 187 78 kDa glucose-regulated protein	Elongation factor 1-alpha merozoite surface protein P12 Putative E3 ubiquitin-protein ligase protein	Heat shock 70 kDa protein	Proliferating cell nuclear antigen
34 kDa/TNF	Fructose-bisphosphate aldolase Aspartic acid-rich prot Plasmepsin-1 Plasmepsin-2 Ornithine aminotransferase	Elongation factor 1-alpha Merozoite surface protein P12 Dynein heavy chain-like protein	Acidic leucine-rich nuclear phosphoprotein 32-related 40S ribosomal protein S3a Proliferating cell nuclear antigen L-lactate dehydrogenase Aspartic acid-rich protein	Glyceraldehyde-3-phosphate dehydrogenase Aspartic acid-rich protein Apolipoprotein E
70 kDa	Knob-associated histidine-rich protein	Reticulocyte-binding protein 3 Dynein heavy chain-like protein Reticulocyte-binding protein 2 MATH and LRR domain-containing protein Putative E3 ubiquitin-protein ligase protein	Knob-associated histidine-rich protein 101 kDa malaria antigen Probable cathepsin C Heat shock 70 kDa protein 78 kDa glucose-regulated protein homologue	Knob-associated histidine-rich protein

## Discussion

The intention of this work was to look for parasite signalling molecules and understand how malaria parasites might sense the host environment. To exist in a wide range of environmental niches, organisms must sense and respond to a variety of external signals.

A primary means by which external sensing occurs in bacteria is through two-component signal transduction pathways, typically composed of a sensor histidine kinase that receives the input stimuli and then phosphorylates a response regulator that effects an appropriate change in cellular physiology. The sensing system described in this paper could be part of a two-component signal transduction pathway in *P. falciparum*, which would be the first time such a system has been recognized and reported. The results indicate that PCNA1 is most likely a component of this sensing system. PCNA1 is an auxiliary protein of DNA polymerase-delta, forms a trimer ring around a DNA double-helix and is a member of the DNA sliding clamp family [37]. It is absent or present in very low amounts in normal non-dividing cells and tissues, but synthesizes in variable amounts by proliferating cells [38, 39].

Phosphorylation/dephosphorylation, ubiquitination, sumoylation, and acetylation have all been described for nuclear PCNA and offer a range of options to modulate PCNA activities [40]. Post-translational modification of cytosolic PCNA has been assumed as a key factor in neutrophil survival. Neutrophils express high levels of PCNA localized exclusively in cytosol, which is ubiquitinated and degraded via the proteasome during apoptosis. Furthermore, PCNA nuclear to cytoplasmic delocalization at the end of granulocyte differentiation has also been demonstrated [41, 42]. These post-translational modifications of PCNA may be crucial in influencing the cellular choice between different pathways, such as the cell cycle checkpoint, DNA repair or apoptosis in order to maintain genomic stability. A recent study has shown that both Pf-PCNA1 and Pf-PCNA2 participate in an active DNA-damage-response pathway with significant accumulation in the parasite upon DNA damage induction, but Pf-PCNA-mediated regulation was not at the level of transcription, but presumably at the protein stability level [43]. It has been suggested by this study that one feature of regulation is via post-translational modification, and that phosphorylation/dephosphorylation controls some functions of Pf-PCNA1.

Tumour necrosis factor (TNF) is a pro-inflammatory cytokine that plays a critical role in diverse cellular events, including cell proliferation, differentiation, and apoptosis. Following stimuli by pro-inflammatory cytokines such as TNF and interleukin-1, IkBs are phosphorylated by IkB kinase (IKK) in mammalian species resulting in their rapid, proteasome-dependent degradation [44, 45]. IkBs are regulatory proteins that inhibit NFkB by complexing with and trapping it in the cytoplasm. They are involved in events including cell adhesion, immune and pro-inflammatory responses, apoptosis, differentiation, and growth. Although this machinery is not thought to exist in *Plasmodium* parasites, an analogous system may have evolved to detect this important host protein. Signal transduction regulation inside *Plasmodium* has been shown as a major mechanism to control parasite development [15]. In the mammalian host, studies have shown that TNF induces extensive alterations in host microvascular endothelium, including morphological re-organization [46], release of membrane microparticles [47], production of other pro-inflammatory cytokines, up-regulation of receptors and apoptosis [48, 49]. Interestingly, inhibitors of TNF production reduced infected erythrocyte cytoadherence [50, 51]. However, TNF signalling in the *Plasmodium* parasite itself has not been previously reported, therefore, understanding the mechanisms of sensing TNF stimulation seen in this study could improve the molecular description of how *Plasmodium* adapts to its host environment.

In a parallel study looking for TNF effects on parasite calcium-dependent signalling, the work showed the ability of the parasite to increase intracellular calcium concentration after exposure to TNF, thus corroborating the hypothesis of an external sensing system allowing the parasite to sense the environment (Cruz et al., unpublished data). This behaviour is also linked to the parasite synchronicity to host circadian rhythms [52] being previously shown that the parasite cell cycle can be modulated by external signals such as melatonin and products of tryptophan catabolism, leading to intracellular calcium increase thought a PLC-IP3 mechanism [53–55]. *Plasmodium* is also able to sense external ATP through rise in cytosolic calcium [15, 56]. Moreover, evidence has also been provided for K^−^/Ca^2+^ signalling mechanisms in *P. falciparum* [57].

The pRBC ghosts contain haemoglobin-depleted erythrocyte material that has lost most of its internal proteins, therefore, this fraction represents an enriched erythrocyte membrane compartment containing molecules attached/associated with the membrane. Parasite-encoded membrane proteins translocated to the surface of infected erythrocytes or in specialized vesicles underneath (Maurer’s clefts), play a key role in the asexual lifecycle. How might all these membrane proteins fit together in the parasite’s ‘sensing apparatus’? Many studies have reported that heat shock proteins HSP70 and HSP90 regulate a number of signalling cascades to maintain cellular homeostasis several species [58, 59]. Recent studies have revealed that HSP70 and HSP90 proteins regulate the function of the IKK complex, which is the major activator of the NF-κB complex [60]. It has been shown that the histidine kinase auto-phosphorylates in the presence of an intra-or extra-cellular environment stimulus or stress, which could be one of the triggers. HSP90 plays an important role in TNF-mediated NF-kB activation by modulating the stability and solubility of receptor interacting protein (RIP) [61]. In this study, antibody AB4 recognized a 90 kDa protein consistent with being HSP90, which was supported by Western blot with an anti-HSP90 antibody (Additional file 4). This was also confirmed by co-immunoprecipitation followed by mass spectrum. HSP90 inhibitor Gambogic acid not only inhibited the expression of this 90 kDa protein, but also abrogated the effects of TNF on PfAB4. This suggests that PfPCNA1 may be one of the HSP90 client proteins, which may explain its identification by AB4 immuno-precipitation and co-migration in the gel electrophoresis. Such an association has been reported previously in several cancer cell lines [62]. The proteins in the insoluble fraction are probably cell membrane components or have high affinity binding to the cytoskeleton, and these potentially could be the ‘perception proteins’ of pRBC. Although data are incomplete they suggest, for the first time, a link between PCNA1 and HSP90 in a parasite sensing system.

An attempt was made to investigate the outcome of the TNF regulatory mechanism of *P. falciparum* parasite strains by analysing transcriptomes of the 3D7 and Dd2 strains (representing high and low PfAB4 phenotypes) with and without TNF treatment by RNAseq analysis. A remarkable conservation of the normal transcriptional programme between these strains has previously been observed [63]. The results showed no transcriptional differences associated with TNF treatment. This suggests that post-transcriptional mechanisms may drive the signalling processes and changes to the global transcription pattern in the parasite may only occur in subsequent cycles, which was not investigated. Indeed it is tempting to speculate that changes in *var* gene expression might be linked to the TNF status in the host to align with altered host adhesion profiles, and might not be detected until one or more cycles after exposure of pRBC to this cytokine.

The cell cycle checkpoints are signal transduction pathways that respond to damaged DNA by inhibiting cell cycle progression [64]. The post-translational modifications of PCNA are considered to be the most relevant during the S phase cell cycle checkpoint (surveillance system) [65]. PCNA interacts with several eukaryotic cell cycle proteins, binds to cyclin-CDK complexes [66] and CDK inhibitor p21 [67, 68]. PCNA forms complexes with critical checkpoint proteins, transducing both positive and negative signals [69]. It is possible that cell cycle checkpoints may serve as switch points for choosing between cell proliferation and apoptosis, or even have a broader role in parasite replication. The ability to sense the external environment and, for example, move a proportion of the population into a quiescent phase could be beneficial to the parasite in avoiding killing by drugs and a protein such as PfPCNA1 could play a significant role in this.

## Conclusion

This study reported a novel sensing system and some potential regulation mechanisms by which parasites respond to external stimuli including inflammatory cytokines, such as TNF. The sensing and regulating system/rapid responding system in *P. falciparum* described here might be an equivalent of the two-component regulatory system of bacterial signal transduction. HSP90 could be a component of a membrane-bound histidine kinase that receives the input stimuli from as yet uncharacterized external receptors, and reversible phosphorylated PfPCNA1 could be a response regulator that effects an appropriate change in cellular physiology from this event. This sensing system might also be shared with cell cycle checkpoints, the stimuli which triggered PCNA1 dephosphorylation/degradation may also be implicated in DNA replication stress and damage. Further work is needed to identify the sensor proteins necessary for interacting with specific external signals and the outcomes for the parasite from this system.

## Additional files

10.1186/s12936-016-1144-6 The proteins from normal RBC and ItG pRBC were extracted and separated by SDS-PAG-electrophoresis. The signalling proteins for ten pathways were measured by Western blot. The red arrow indicates the only positive identification of PfAB4. The experiment has been performed independently three times to confirm the results.
10.1186/s12936-016-1144-6 The osmolality of culture reagents used in the laboratory were measured using a pocket OSMOCHECK (PAL-OSMO) (VITECH Scientific Ltd, UK) (A). ItG-pRBC growing in blue (serum containing) medium were placed into Plasmion mix (1:1.5:2.5) for 20 min. Then these pRBCs were centrifuged to change to either blue or yellow (serum-free) medium for 5, 15 and 60 min. The expression of PfAB4 was measured by Western blot (B). The results indicated that transfer from lower osmolality solution to a higher osmolality (blue to 1:1.5:2.5) had no effect on expression of PfAB4, but from higher osmolality solution to a lower osmolality solution (1:1.5:2.5 to blue or yellow) increased the expression of PfAB4.
10.1186/s12936-016-1144-6 The proteins from Dd2 and ItG pRBC were extracted at the time points of IDC and separated by SDS-PAG-electrophoresis, followed by Western blot using AB4 antibodies. The results show that the expression of PfAB4 in Dd2 was low (A) and the protein was localized in the cytosol (B).
10.1186/s12936-016-1144-6 ItG-pRBC were treated with 10 ng/ml TNF for 60, 30, 15 and 5 min and the cellular proteins were extracted and separated by SDS-PAGE, followed by Western blot probed with anti-HSP90 and AB4 antibodies. The 90kDa protein recognized by the two antibodies showed similar expression patterns.
