# Supplementary figures and images for: An external sensing system in Plasmodium falciparum-infected erythrocytes
Source: Malar J. 2016 Feb 19;15:103. doi: 10.1186/s12936-016-1144-6 (PMC4759932; doi:10.1186/s12936-016-1144-6)

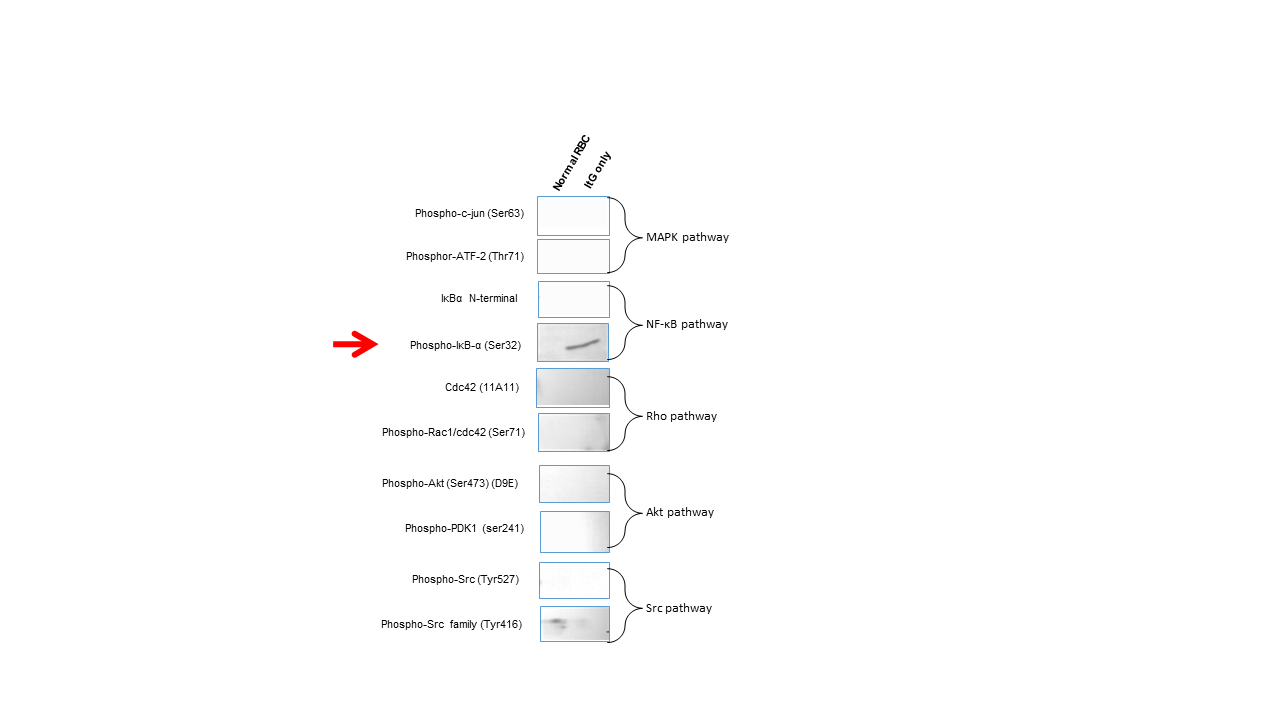

Supplement: Supplementary file 1 — 10.1186/s12936-016-1144-6 The proteins from normal RBC and ItG pRBC were extracted and separated by SDS-PAG-electrophoresis. The signalling proteins for ten pathways were measured by Western blot. The red arrow indicates the only positive identification of PfAB4. The experiment has been performed independently three times to confirm the results. [file 12936_2016_1144_MOESM1_ESM.tif]

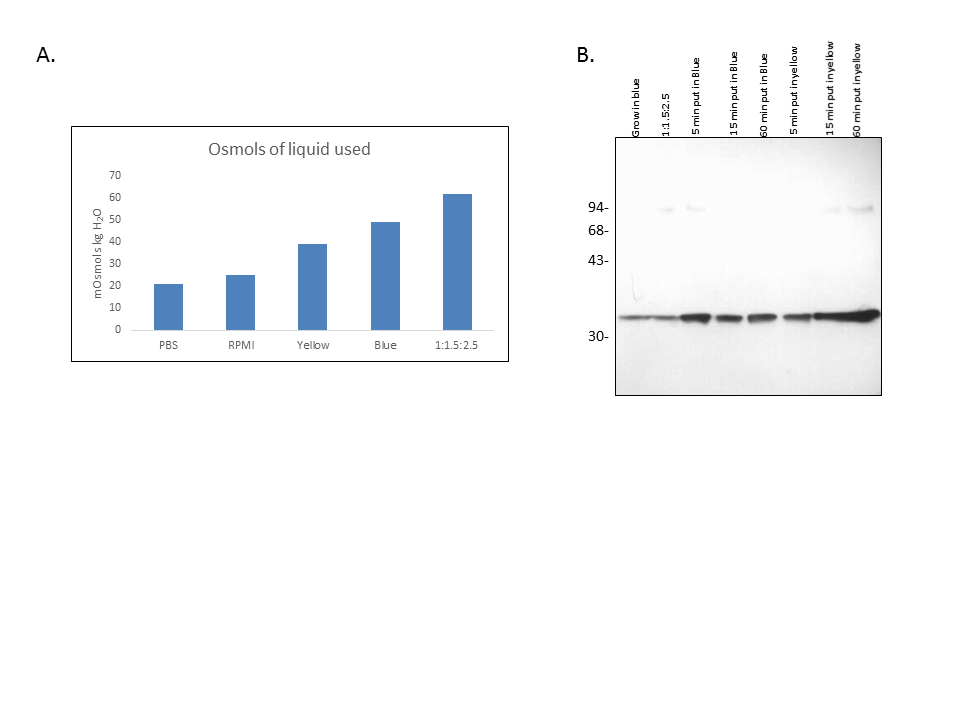

Supplement: Supplementary file 2 — 10.1186/s12936-016-1144-6 The osmolality of culture reagents used in the laboratory were measured using a pocket OSMOCHECK (PAL-OSMO) (VITECH Scientific Ltd, UK) (A). ItG-pRBC growing in blue (serum containing) medium were placed into Plasmion mix (1:1.5:2.5) for 20 min. Then these pRBCs were centrifuged to change to either blue or yellow (serum-free) medium for 5, 15 and 60 min. The expression of PfAB4 was measured by Western blot (B). The results indicated that transfer from lower osmolality solution to a higher osmolality (blue to 1:1.5:2.5) had no effect on expression of PfAB4, but from higher osmolality solution to a lower osmolality solution (1:1.5:2.5 to blue or yellow) increased the expression of PfAB4. [file 12936_2016_1144_MOESM2_ESM.tif]

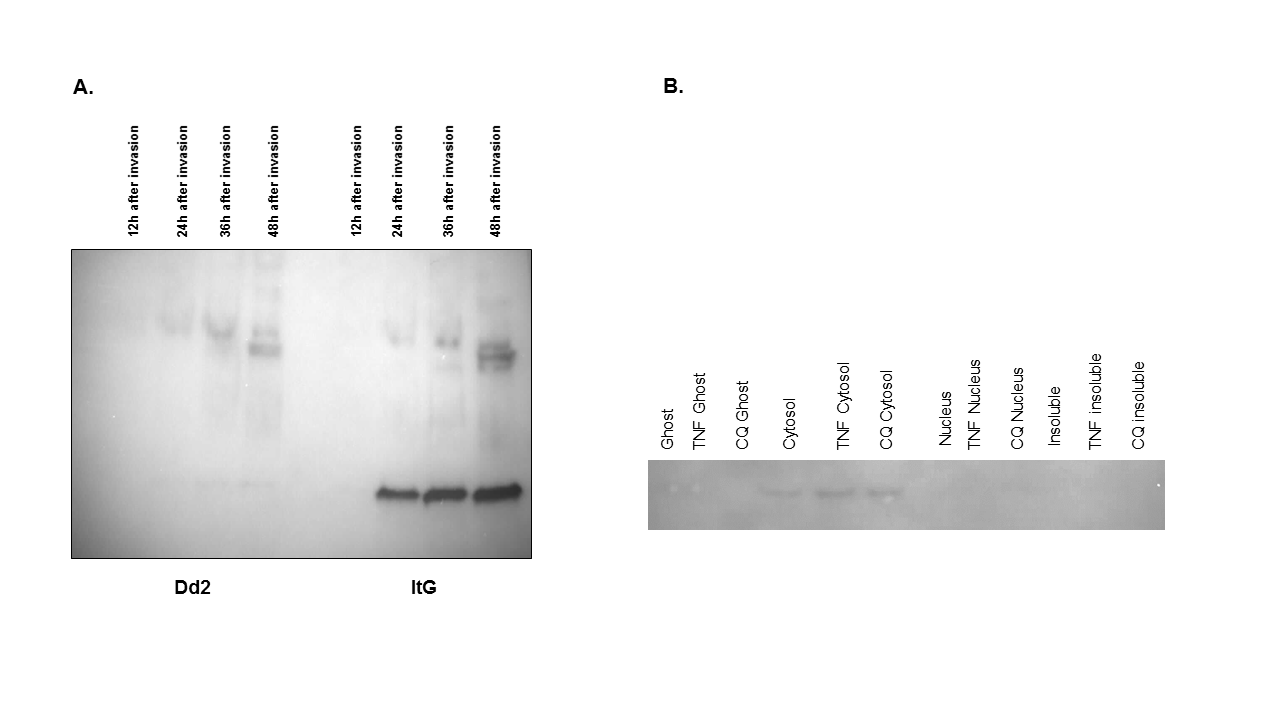

Supplement: Supplementary file 3 — 10.1186/s12936-016-1144-6 The proteins from Dd2 and ItG pRBC were extracted at the time points of IDC and separated by SDS-PAG-electrophoresis, followed by Western blot using AB4 antibodies. The results show that the expression of PfAB4 in Dd2 was low (A) and the protein was localized in the cytosol (B). [file 12936_2016_1144_MOESM3_ESM.tif]

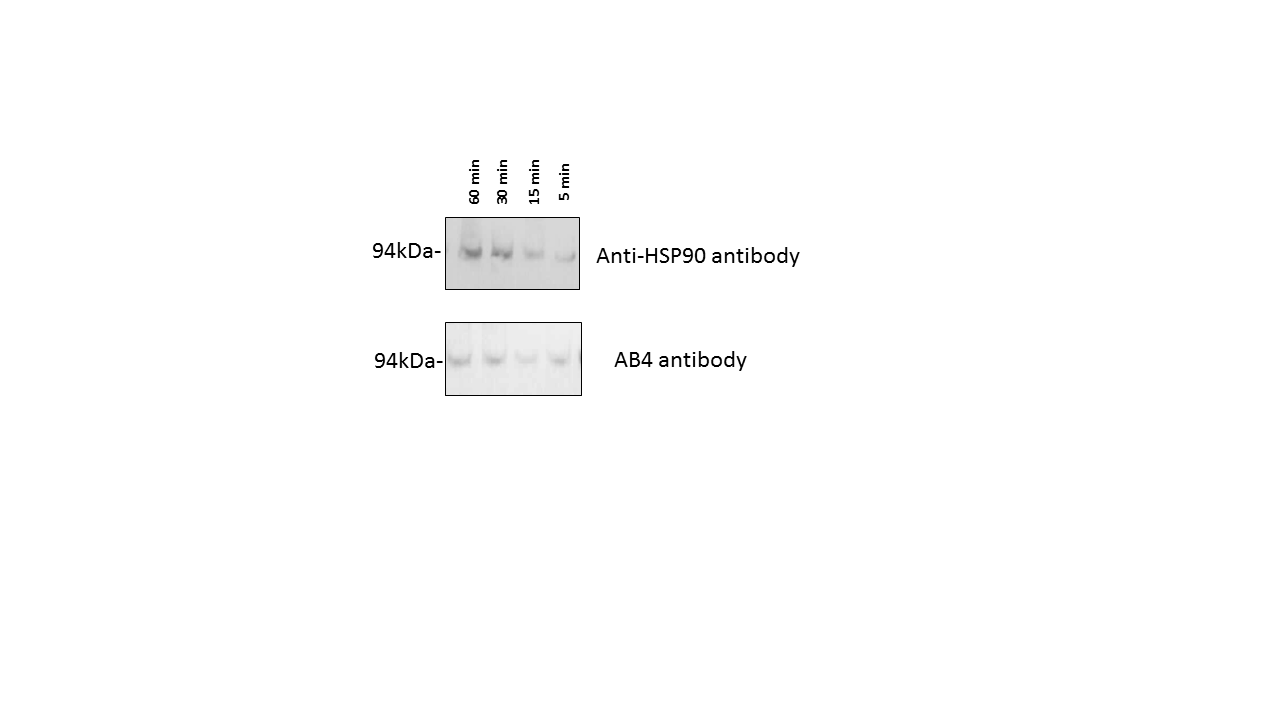

Supplement: Supplementary file 4 — 10.1186/s12936-016-1144-6 ItG-pRBC were treated with 10 ng/ml TNF for 60, 30, 15 and 5 min and the cellular proteins were extracted and separated by SDS-PAGE, followed by Western blot probed with anti-HSP90 and AB4 antibodies. The 90kDa protein recognized by the two antibodies showed similar expression patterns. [file 12936_2016_1144_MOESM4_ESM.tif]
